# Supplementary material for: Stabilization of body balance with Light Touch following a mechanical perturbation: Adaption of sway and disruption of right posterior parietal cortex by cTBS
Source: PLoS One. 2020 Jul 2;15(7):e0233988. doi: 10.1371/journal.pone.0233988 (PMC7332304; doi:10.1371/journal.pone.0233988)
Supplement: S1 File — (DOCX) [file pone.0233988.s001.docx]

**Linear Model Estimates**

1. **LMM**

Variable~Stimulation_Session+Hand_Contact+Test+Force_Push+Stimulation_Session*Hand_Contact+Stimulation_Session*Test+Stimulation_Session*Force_Push+Hand_Contact*Test+LT*Force_Push+Test*Force_Push+Stimulation_Session*Hand_Contact*Test+Stimulation_Session*Test*Force_Push+Stimulation_Session*Hand_Contact*Force_Push+Hand_Contact*Test*Force_Push+Stimulation_Session*Hand_Contact*Test*Force_Push + (1 |Subjects)

Intercept

Fixed effects:

Estimate Std. Error t value

(Intercept) 1.541216 0.046169 33.382

cTBSstim -0.083649 0.055516 -1.507

LTtouch -0.089506 0.052854 -1.693

testpre 0.081747 0.052854 1.547

force 0.074972 0.007968 9.409

cTBSstim:LTtouch 0.025936 0.077518 0.335

cTBSstim:testpre 0.083192 0.076652 1.085

cTBSstim:force 0.014068 0.011717 1.201

LTtouch:testpre -0.094035 0.074747 -1.258

LTtouch:force 0.011722 0.011269 1.040

testpre:force -0.006940 0.011269 -0.616

cTBSstim:LTtouch:testpre -0.026723 0.107747 -0.248

cTBSstim:testpre:force -0.011722 0.016256 -0.721

cTBSstim:LTtouch:force -0.001980 0.016451 -0.120

LTtouch:testpre:force 0.013083 0.015936 0.821

cTBSstim:LTtouch:testpre:force -0.001106 0.022975 -0.048

Constant

Fixed effects:

Estimate Std. Error t value

(Intercept) 0.7458996 0.0658653 11.325

cTBSstim 0.0068594 0.0656921 0.104

LTtouch -0.0771862 0.0625373 -1.234

testpre 0.0431729 0.0625373 0.690

force 0.0127375 0.0094278 1.351

cTBSstim:LTtouch -0.0522433 0.0917216 -0.570

cTBSstim:testpre 0.1213881 0.0906992 1.338

cTBSstim:force 0.0091007 0.0138640 0.656

LTtouch:testpre -0.0880186 0.0884410 -0.995

LTtouch:force -0.0017114 0.0133330 -0.128

testpre:force 0.0163712 0.0133330 1.228

cTBSstim:LTtouch:testpre -0.0106900 0.1274878 -0.084

cTBSstim:testpre:force -0.0358592 0.0192349 -1.864

cTBSstim:LTtouch:force 0.0079257 0.0194645 0.407

LTtouch:testpre:force 0.0064127 0.0188557 0.340

cTBSstim:LTtouch:testpre:force 0.0006087 0.0271849 0.022

EMG Integral Tibialis Anterior

Fixed effects:

Estimate Std. Error t value

(Intercept) 0.248553 0.015878 15.654

cTBSstim 0.006866 0.014677 0.468

LTtouch -0.013533 0.012457 -1.086

testpre -0.004272 0.012457 -0.343

force 0.001484 0.001878 0.790

cTBSstim:LTtouch 0.012445 0.020177 0.617

cTBSstim:testpre 0.050924 0.019781 2.574

cTBSstim:force -0.001545 0.003058 -0.505

LTtouch:testpre 0.013843 0.017617 0.786

LTtouch:force 0.002134 0.002656 0.804

testpre:force 0.003439 0.002656 1.295

cTBSstim:LTtouch:testpre -0.030507 0.027629 -1.104

cTBSstim:testpre:force -0.003466 0.004173 -0.831

cTBSstim:LTtouch:force -0.001700 0.004262 -0.399

LTtouch:testpre:force -0.004398 0.003756 -1.171

cTBSstim:LTtouch:testpre:force 0.002866 0.005889 0.487

EMG Integral Gastrocnemius

Fixed effects:

Estimate Std. Error t value

(Intercept) 0.3010887 0.0098112 30.688

cTBSstim 0.0093315 0.0108806 0.858

LTtouch -0.0260647 0.0092360 -2.822

testpre 0.0040091 0.0092360 0.434

force 0.0004338 0.0013924 0.312

cTBSstim:LTtouch 0.0156623 0.0149601 1.047

cTBSstim:testpre 0.0080756 0.0146659 0.551

cTBSstim:force -0.0009498 0.0022671 -0.419

LTtouch:testpre 0.0027786 0.0130617 0.213

LTtouch:force 0.0024295 0.0019691 1.234

testpre:force 0.0001192 0.0019691 0.061

cTBSstim:LTtouch:testpre -0.0070573 0.0204851 -0.345

cTBSstim:testpre:force 0.0005407 0.0030937 0.175

cTBSstim:LTtouch:force -0.0025401 0.0031602 -0.804

LTtouch:testpre:force -0.0012266 0.0027848 -0.440

cTBSstim:LTtouch:testpre:force 0.0017121 0.0043661 0.392

EMG Integral Soleus

Fixed effects:

Estimate Std. Error t value

(Intercept) 167.2441 47.8491 3.495

cTBSstim 8.1194 26.7247 0.304

LTtouch -19.7197 22.6796 -0.869

testpre 46.3222 22.6796 2.042

force -0.1258 3.4191 -0.037

cTBSstim:LTtouch 14.2454 36.7364 0.388

cTBSstim:testpre -40.2275 36.0104 -1.117

cTBSstim:force -0.9002 5.5672 -0.162

LTtouch:testpre 2.9430 32.0738 0.092

LTtouch:force 1.8213 4.8353 0.377

testpre:force -7.7909 4.8353 -1.611

cTBSstim:LTtouch:testpre -1.4060 50.2998 -0.028

cTBSstim:testpre:force 8.4788 7.5969 1.116

cTBSstim:LTtouch:force -0.8254 7.7601 -0.106

LTtouch:testpre:force -0.0167 6.8382 -0.002

cTBSstim:LTtouch:testpre:force -0.9898 10.7213 -0.092

EMG Peak Activity Tibialis Anterior

Fixed effects:

Estimate Std. Error t value

(Intercept) -1.25945 0.13468 -9.352

cTBSstim 0.15361 0.16207 0.948

LTtouch -0.14494 0.13662 -1.061

testpre -0.16558 0.13662 -1.212

force 0.02507 0.02110 1.188

cTBSstim:LTtouch 0.07813 0.21848 0.358

cTBSstim:testpre 0.26640 0.21434 1.243

cTBSstim:force -0.01403 0.03541 -0.396

LTtouch:testpre 0.19766 0.18994 1.041

LTtouch:force 0.02085 0.02900 0.719

testpre:force 0.05309 0.02900 1.831

cTBSstim:LTtouch:testpre -0.15960 0.29617 -0.539

cTBSstim:testpre:force -0.05262 0.04646 -1.133

cTBSstim:LTtouch:force -0.01543 0.04736 -0.326

LTtouch:testpre:force -0.05643 0.04040 -1.397

cTBSstim:LTtouch:testpre:force 0.03895 0.06400 0.609

EMG Peak Activity Gastrocnemius

Fixed effects:

Estimate Std. Error t value

(Intercept) -1.076374 0.077412 -13.905

cTBSstim -0.130374 0.073269 -1.779

LTtouch -0.130331 0.061746 -2.111

testpre 0.034495 0.061746 0.559

force -0.009302 0.009535 -0.976

cTBSstim:LTtouch 0.069032 0.098744 0.699

cTBSstim:testpre 0.130533 0.096873 1.347

cTBSstim:force 0.021242 0.016005 1.327

LTtouch:testpre 0.028089 0.085840 0.327

LTtouch:force 0.018384 0.013105 1.403

testpre:force 0.007288 0.013105 0.556

cTBSstim:LTtouch:testpre -0.093538 0.133848 -0.699

cTBSstim:testpre:force -0.011635 0.020997 -0.554

cTBSstim:LTtouch:force -0.027218 0.021406 -1.272

LTtouch:testpre:force -0.017185 0.018259 -0.941

cTBSstim:LTtouch:testpre:force 0.022665 0.028923 0.784

EMG Peak Activity Soleus

Fixed effects:

Estimate Std. Error t value

(Intercept) -1.153282 0.125213 -9.211

cTBSstim -0.091361 0.088323 -1.034

LTtouch -0.198012 0.074423 -2.661

testpre -0.067774 0.074423 -0.911

force -0.010430 0.011493 -0.908

cTBSstim:LTtouch 0.111054 0.119019 0.933

cTBSstim:testpre 0.079351 0.116764 0.680

cTBSstim:force 0.007984 0.019291 0.414

LTtouch:testpre 0.107350 0.103463 1.038

LTtouch:force 0.024241 0.015795 1.535

testpre:force 0.007018 0.015795 0.444

cTBSstim:LTtouch:testpre -0.060735 0.161329 -0.376

cTBSstim:testpre:force 0.009222 0.025308 0.364

cTBSstim:LTtouch:force -0.012272 0.025801 -0.476

LTtouch:testpre:force -0.019983 0.022007 -0.908

cTBSstim:LTtouch:testpre:force -0.002543 0.034862 -0.073

1. **LMM:**

Variable~Stimulation_Session+Hand_Contact+Block+Stimulation_Session*Hand_Contact+Stimulation_Session*Block+Stimulation_Session+Hand_Contact*Block+LT+Block+Stimulation_Session*Hand_Contact*Block+Stimulation_Session*Block+Stimulation_Session*Hand_Contact+Hand_Contact*Block+Stimulation_Session*Hand_Contact*Block + (1 |Subjects)

Intercept

Fixed effects:

Estimate Std. Error t value

(Intercept) 97.5960 6.8633 14.220

cTBSstim 3.1312 7.1785 0.436

LTtouch -10.7821 7.6441 -1.411

Time -1.3601 0.6534 -2.082

cTBSstim:LTtouch -10.0260 10.8164 -0.927

cTBSstim:Time 0.2315 0.9387 0.247

LTtouch:Time 0.7004 1.0492 0.668

cTBSstim:LTtouch:Time 0.8352 1.4950 0.559

Constant

Fixed effects:

Estimate Std. Error t value

(Intercept) 10.736641 1.047459 10.250

cTBSstim -0.464469 0.806987 -0.576

LTtouch -3.237307 0.859312 -3.767

Time -0.292079 0.073453 -3.976

cTBSstim:LTtouch -0.301011 1.215954 -0.248

cTBSstim:Time 0.128511 0.105531 1.218

LTtouch:Time 0.163520 0.117944 1.386

cTBSstim:LTtouch:Time -0.008463 0.168059 -0.050

Slope

Fixed effects:

Estimate Std. Error t value

(Intercept) -0.7594 28.5032 -0.027

cTBSstim -6.0703 40.7556 -0.149

LTtouch 2.3150 43.4122 0.053

Time -7.4819 3.7108 -2.016

cTBSstim:LTtouch -13.4243 61.4005 -0.219

cTBSstim:Time 7.6873 5.3281 1.443

LTtouch:Time 6.6081 5.9585 1.109

cTBSstim:LTtouch:Time -5.0579 8.4873 -0.596

EMG Integral Tibialis Anterior

Fixed effects:

Estimate Std. Error t value

(Intercept) 78.0321 16.0479 4.862

cTBSstim 39.2004 10.2409 3.828

LTtouch -13.0185 10.0860 -1.291

Time -1.8169 0.8621 -2.107

cTBSstim:LTtouch -7.3374 15.2377 -0.482

cTBSstim:Time -3.5640 1.3374 -2.665

LTtouch:Time 1.5010 1.3843 1.084

cTBSstim:LTtouch:Time 0.4393 2.1203 0.207

EMG Integral Gastrocnemius

Fixed effects:

Estimate Std. Error t value

(Intercept) 115.409 14.264 8.091

cTBSstim 14.061 12.500 1.125

LTtouch -27.240 12.364 -2.203

Time -1.085 1.057 -1.026

cTBSstim:LTtouch 24.058 18.647 1.290

cTBSstim:Time -1.416 1.635 -0.866

LTtouch:Time 1.288 1.697 0.759

cTBSstim:LTtouch:Time -1.625 2.596 -0.626

EMG Integral Soleus

Fixed effects:

Estimate Std. Error t value

(Intercept) 187.4139 42.9060 4.368

cTBSstim -10.3742 15.2184 -0.682

LTtouch -16.7387 14.9360 -1.121

Time -2.6609 1.2767 -2.084

cTBSstim:LTtouch 15.8327 22.5977 0.701

cTBSstim:Time 1.5936 1.9848 0.803

LTtouch:Time 1.0000 2.0500 0.488

cTBSstim:LTtouch:Time -0.7696 3.1426 -0.245

EMG Peak Activity Tibialis Anterior

Fixed effects:

Estimate Std. Error t value

(Intercept) -1.069570 0.108734 -9.837

cTBSstim 0.140155 0.087611 1.600

LTtouch -0.076942 0.085149 -0.904

Time -0.005446 0.007546 -0.722

cTBSstim:LTtouch -0.020347 0.129143 -0.158

cTBSstim:Time -0.008225 0.011871 -0.693

LTtouch:Time -0.001001 0.011822 -0.085

cTBSstim:LTtouch:Time 0.008017 0.018239 0.440

EMG Peak Activity Gastrocnemius

Fixed effects:

Estimate Std. Error t value

(Intercept) -1.0532657 0.0705454 -14.930

cTBSstim 0.0581472 0.0527651 1.102

LTtouch -0.0847062 0.0512799 -1.652

Time -0.0055995 0.0045445 -1.232

cTBSstim:LTtouch -0.0327581 0.0777750 -0.421

cTBSstim:Time -0.0129795 0.0071494 -1.815

LTtouch:Time 0.0027636 0.0071195 0.388

cTBSstim:LTtouch:Time -0.0007288 0.0109840 -0.066

EMG Peak Activity Soleus

Fixed effects:

Estimate Std. Error t value

(Intercept) -1.211986 0.118068 -10.265

cTBSstim 0.042914 0.067240 0.638

LTtouch -0.071864 0.065340 -1.100

Time -0.002785 0.005791 -0.481

cTBSstim:LTtouch -0.004256 0.099100 -0.043

cTBSstim:Time -0.007882 0.009110 -0.865

LTtouch:Time 0.001026 0.009072 0.113

cTBSstim:LTtouch:Time 0.005013 0.013996 0.358
