# Supplementary material for: Stabilization of body balance with Light Touch following a mechanical perturbation: Adaption of sway and disruption of right posterior parietal cortex by cTBS
Source: PLoS One. 2020 Jul 2;15(7):e0233988. doi: 10.1371/journal.pone.0233988 (PMC7332304; doi:10.1371/journal.pone.0233988)
Supplement: Attachment — Submitted filename: Response to Reviewers.docx [file pone.0233988.s002.docx]

Dear Editor,

we would like to thank the reviewers for their very valuable criticisms and comments regarding our study “Stabilization of body balance with light touch following a mechanical perturbation: Adaption of sway and disruption of right Posterior Parietal Cortex by cTBS”. We followed the reviewers’ advice and revised our manuscript thoroughly. Below we explain the changes made to the manuscript point-by-point.

**Reviewer 1**

1. The task is correctly described but unless I missed it, there was no description of the instructions given to the subject. This is important as it can obviously affect their behaviour following the robotic push (e.g. “resist the push” vs. “stand still” vs. “limit your centre of mass movements”).

We added information regarding the instructions given to the subject. Subject were merely instructed to stand as natural as possible, in order to avoid making the task of maintaining balance explicit (Line 184).

2. I praise the authors for controlling stimulation site with both neuronavigation and anatomical support. However, the description of the protocol needs to be more detailed. cTBS intensity relies on the correct assessment of a motor threshold (active or passive depending on the literature). Please explain how the motor threshold was obtained, and in which muscle. Please comment on the fact that the MT was assessed in a place different than the stimulation site (if I understand correctly). How the authors can be certain that the MT would be the same at different cortical position (given the possible differences in axons orientation due to possibly different cortical folding)? Moreover, also possibly due to a difference in cells orientation but also in activity, the region stimulated may not respond to cTBS the same way than the FDI motor region. See for example https://doi.org/10.1016/j.clinph.2006.08.008. Further, you have not tested whether the cTBS stimulation was successful at disrupting the area. This is a big concern since it has been shown that cTBS has extremely variable effects (10.1016/j.clinph.2017.08.023). Therefore, in the present experiment, we do not know whether cTBS was able to disrupt the area, or if it did it whether it increased or decreased the region excitability. Please address these concerns in a limitation section and change your discussion accordingly. What was the procedure for the sham stimulation?

We added additional information about the procedure of assessing the motor threshold (Line 237). We also added from which muscle it was measured. Given we implemented and followed standard procedure, we are positive that MT was determined correctly. The procedure served to individually standardize the stimulation strength since important factors such as thickness of the scull bone vary inter-individually more than across different cortical areas of an individual person. Stimulation of non-motor cortical areas with a fixed strength determined by M1 stimulation is a frequently used method (Siebner & Ziemann, 2007). We are however aware, that like with all such TMS protocols applied outside primary motor regions there is a degree of uncertainty regarding the effectives. Observed effects of stimulation seem likely to stem from inhibition of the cortical target area, given that the sham coil is not able to produce and thus induce cortical changes. Behavioral changes observed seem thus likely to be the result of disruption of the rPPC. We added information regarding the sham coil as well (Line 247). However, as the reviewer suggested, we added a limitation section pointing out that we were not able to assess whether excitability of the target area was indeed reduced. It is fair to point out, that due to the nature of the rPPC a direct assessment, which does not include fMRI or EEG, of a change of excitability is simply not possible.

3. Statistics. I salute the authors for using LMM. However, the statistical part is not detailed enough to understand how statistics were performed and how the readers should interpret results. What are the distributions of your data sets? If they are not normal, please at least indicate it to the reader (in the methods and in a limitation section), and data transformation or use of GLM is advised. Please details the models used: Are the 4 fixed effects implemented in the same model or in different models? How are the models hierarchized and the data clustered? What are the different levels within the factors (are they treated as factor or as continuous variable)? What are the random effects? In the statistic section it reads that there was no interaction between factors in the model, but then in the results it seems that there were interactions. Furthermore, it seems that the model was not fully “maximal” according to errors, which make it more prone to type I error (see Barr 2013). Please maximize the error structure including at the interaction level. How did you obtain p-values with the LMM, have you used the LmerTest package? You have performed post-hoc analysis. Please describe them. Given the model you used, these post hoc tests may not be necessary when looking directly at the models parameters. In the Tables, in the title line, there is a F(). What does it stand for? F-values? Adding parameters values (intercepts and betas) and their CI or errors would help the comprehension of the tables.

We followed the advice of the reviewer and substantially revised our statistics section. In addition we firstly introduced a cluster analysis to be certain to identify possible non-responder to the stimulation. Data was checked for normal distribution. Since it did not meet normal distribution it was then log-transformed and LMM was applied again. Tables in the result section and the method section have been corrected accordingly. We also provided more detailed information regarding the used models. We also improved the model and maximized it. Used R-package for data analysis has also been added as information in the method section (Line 301 to 325).

4. In the Tables, what is the factor Test?

Factor Test stands for the evaluation of pre- vs. post-stimulation. Method section was corrected accordingly. In the Methods section this factor was previously named “effect of stimulation”, this has been corrected and “Test” is now used overall (Line 314).

5. What is the rationale behind separating analysis for peak EMG activity and EMG activity. How should we interpret a change in Peak activity but no change in general activity? This separation increases the number of tests performed, which in a frequentist paradigm increases the risks of false alarm (a change in peak but not in general activity and vice-versa seems actually to be more related to a false alarm than a solid physiological result).

General EMG activity and peak EMG activity describes two different factors for stabilization of balance. The peak EMG activity is the highest peak after the perturbation, usually indicative of the immediate perturbation response. The Integral of the EMG (general muscle activity) describes the tonic activation of the muscle throughout the whole trial (Line 291 – 294). We therefore considered general EMG activity an peak activity as independent measures.

6. In page 19, the authors talk about the lack of effect from the rPPC disruption and how it creates a conundrum with the existing literature. The physiological interpretations should be toned down since there was here no controls of the disruption effectiveness of the cTBS (associated with a low sample size). The authors should also discuss the fact that since they have not controlled for the effectiveness of the cTBS, the absence of effect is in no way conclusive in regard to the role of the rPPC in regard to posture stabilisation with/without light touch. Using the changes in EMG as a control of the effect of cTBS would not be very credible as well (see comments below).

We added a limitation section to the discussion, discussing the possibility that cTBS did not alter cortical activity of the target area (see point 2) (Line 626 – 646).

7. The authors states that cTBS may have reduced more Muscle activity after perturbation. However, if I read correctly the figure 7 (for the tibialis), the most extreme difference of EMG activity between the 2 sessions occurs before the cTBS stimulation (unless there is a problem in the legends?). I cannot assess whether this observation is similar for the peak activity of GAS since there is no related figure. Therefore, it seems probable to me that the difference observed is more due to variability than from an effect of cTBS. This point is supported by the fact that you obtained a significant interaction between blocks and stimulation only for general activity of for the peak activity but never for both at the same time (while we would expect it to happen, unless I am mistaken about the interpretation of such parameters). In any cases, the observed difference of 5% between sessions seems meaningless compared to the overall observed variability. Please comment this point and change the discussion/abstract/conclusion accordingly.

First of all, we like to mention that due to the log transformation and the maximization of the model statistical outcomes have changed slightly. We now see a stimulation effect in both peak and general EMG activity. It is correct that it seems like the effect stems from the difference between the 2 sessions before cTBS stimulation. It is also correct, that there is a difference between the pre-tests of the two stimulation session. However, this general difference and activation amplitude can result from the placement of the EMG-sensor, which might have deviated slightly in these two sessions. Therefore, it is important to look at the stimulation effect in the two sessions separately. In order to clarify this effect, we carried out another post hoc analysis of the two session separated from another, looking at the mere pre vs. post stimulation effect. We see that there is no significant change of muscle activity following sham stimulation, while we observe a significant change after cTBS stimulation. Since the sham coil is not able to change cortical activity, it seems likely that the observed changes during the cTBS session are in fact a result of altered cortical activity of the rPPC. In order to visualize this better, we changed figure 7 and figure 8, displaying the change of percentage from the first block to the last.

8. In the abstract and in the conclusion the authors indicate that cTBS affected gastrocnemius activity, but in the discussion above it is said that cTBS changed Tibialis activity (p23) or all muscle activity according to the Tables. Maybe some precision should be added there?

We realized a mistake in the conclusion as pointed out by the reviewer. We corrected the conclusion accordingly (Line 664), the description in the discussion and in the tables was correct.

9. In the figure 7, the dashed or solid lines are not following what the legend says.

Figure 7 has been modified. It now displays the percentages of change from the first block to the last as a function of contact condition (Light Touch vs. No Touch) and stimulation protocol (sham vs. cTBS). The same was done for Fig. 6. We believe this kind of display is a more fitting representation of the gradual decrease over time.


**Reviewer 2**

1. The data-analysis process appears quite arbitrary and complicated while there is no obvious reason for some steps of this approach. Specifically, the process of binning and then fitting, as well as the reason for excluding bad fits should be – at least - clarified and justified.

Justification for data analysis process has been added to the method section (Line 275). The same procedure was used in a previous paper by Johannsen et al. 2007. We applied the same procedure in order to make results easier to compare, since the previous study also investigated the effects of light touch for the control of balance following perturbations.

2. Fig. 7: I guess that colors and line style are wrong. Otherwise the results would make absolutely no sense.

Fig. 7 has been corrected. However, it now displays the percentages of change from the first block to the last as a function of contact condition (Light Touch vs. No Touch) and stimulation protocol (sham vs. cTBS). The same was done for Fig. 6. We believe this kind of display is a more fitting representation of the gradual decrease over time and the stimulation effect.

3. Conclusions: “However, we did found an unexpected effect of cTBS stimulation in terms of improvements of the aforementioned adaptive process. After disruption of the rPPC muscle activity of the Gastrocnemius is decreased even greater, compared to sham. From that we can conclude that rPPC disruption enhanced the intra-session adaptation of the disturbing effects of the perturbation.”
   Maybe I missed something, but I am not convinced that this statement is supported by the data.

In response to an advice of the first reviewer we introduced log transformation of the data and the maximization of the model (Line 311), statistical outcomes have changed slightly (See Result Tables). We now see a stimulation effect in both peak and general EMG activity, giving more support to our statement. Furthermore, we added another post hoc analysis of the two session separated from another, looking at the mere pre vs. post stimulation effect. We see that there is no significant change of muscle activity following sham stimulation, while we observe a significant change after cTBS stimulation.

4. General comment: there appear to be several citation styles and inconsistencies in the manuscript. E.g. in line 67 „Dickstein and colleagues (2003) [3]“. So the references should be cleaned up throughout the manuscript.

References have been cleaned up throughout the manuscript.

5. Figure numbers are not correct.
   In Figure 2 (as indicated on the figure itself) also the push conditions could be displayed. Also adding the scheme in Figure 1 into this figure might be possible and would the number of figures.

Figure numbers have been corrected. Push condition cannot be displayed in Figure 2, since forces were always randomized and we wanted to avoid making the impression forces push conditions were the same in every trial of every participant.

6. Line 34: The sentence appears to have some words missing „has been improve...“

Sentence has been corrected.

7. Line 37-40: Are these two hypotheses related to each other? If yes, please clarify. If not, I would suggest to formulate them as two hypotheses and not connect them using ‚but‘.

Hypotheses have been clarified as two hypotheses and abstract corrected accordingly (Line 39 – 43) It now reads: “We hypothesized that the benefit of light touch would be amplified in the more dynamic context of an external perturbation, reducing body sway and muscle activations before, at and after a perturbation. Furthermore we expected sway stabilization would be impaired following disruption of the right Posterior Parietal Cortex as a result of increased postural stiffness.”

8. Line 49: Here you state that „We were not able to confirm our hypothesis that disruption of the rPPC leads to increased postural stiffness.“ However, above you state that: „[…] sway stabilization would be generally impaired following disruption of the right Posterior Parietal Cortex.“
   Maybe its better to state the same thing in the hypothesis formulation and in the results.

We thank the reviewer for pointing out this inconsistency. We corrected this inconsistency in the hypothesis formulation as stated in the previous point.

9. Line 136: The formulation of the hypotheses are a little cryptic. For example: what is meant by “relative benefit [… is] amplified”? Or the “[reduced] facilitation of sway stabilization”. Something closer to the parameters (e.g. reduced sway response) would be much more specific and easier to understand.

Formulation of hypothesis has been clarified and is more consistent with the hypothesis formulation in the abstract. It now reads: “We hypothesized that the benefit of light touch would be amplified in the more dynamic context of an external perturbation to balance, improving the compensation response. We also expected that the immediate response to a perturbation and sway stabilization in terms of its time constant would be affected expressing an increase in postural stiffness following rPPC disruption.” (Line 161 - 166)

10. Line 160: referent should be references

Word has been corrected.

11. Table 1: I don’t believe this table is necessary. If its left in the manuscript a more precise tiltle is required (“… for one example subject”)

We left the table in the manuscript. We believe it is important for readers to know what force the participants were exposed to on average. We did formulate a more precise title.

12. line 217: “CoP position was differentiated to obtain CoP rate-of-change in seconds (dCoP).” Differentiating a position gives velocity – m/s and not s.

The reviewer is correct. “s” has been corrected to “m/s”.

13. line 219: “before and after at the” delete at

“at” was deleted.

14. Figure 3: x-axis labels are missing and x-axis should be given in seconds.

x-axis labels were added.

15. Line numbers are missing starting at page 14.

Line numbers have been added.

16. Page 14 bottom: weather should be whether

Word has been corrected.

17. Fig. 6: legend for different colors are missing.

Fig 6. Has been modified and legend was added.

18. Fig. 7 and 8: I suggest to rename “general muscle activity” by something like “EMG integral”, since the current label is very confusing.

As suggested we renamed “general muscle activity” to “EMG Integral”.

19. Page 17: the introduction of the discussion is much better in clarifying the hypotheses as compared to Abstract and Introduction. Maybe the latter could be reformulated along those lines.

We now introduce the hypotheses more clearly in the abstract and in the introduction and more along the line as in the introduction of the discussion (Line 39 – 34 and Line 161 – 166).

20. Page 22: “cuing or hether they” is this supposed to mean whether?

This word was supposed to mean whether. Word has been corrected.

21. Page 23: “asymptote, with a decrease of 15% in both the 1% and 4% force push condition and 20%” should be “1% and 7%”

There was a mistake with the percentages. This has been corrected. In order to make it easier understandable we changed the wording. 1% is now called small perturbation, 4% is called medium perturbation and 7% is called strong perturbation. Manuscript has been changed accordingly.

22. Page 31: “However, we did found an unexpected effect of cTBS stimulation in terms of improvements of the aforementioned adaptive process.” Should be “we did find”.

Wording has been corrected.
