# Supplementary material for: Stabilization of body balance with Light Touch following a mechanical perturbation: Adaption of sway and disruption of right posterior parietal cortex by cTBS
Source: PLoS One. 2020 Jul 2;15(7):e0233988. doi: 10.1371/journal.pone.0233988 (PMC7332304; doi:10.1371/journal.pone.0233988)
Supplement: Attachment — Submitted filename: Response to Reviewers.docx [file pone.0233988.s003.docx]

Dear Editor,

we would like to thank the reviewers for their very valuable criticisms and comments regarding our study “Stabilization of body balance with light touch following a mechanical perturbation: Adaption of sway and disruption of right Posterior Parietal Cortex by cTBS”. We followed the reviewers’ advice and revised our manuscript thoroughly. Below we explain the changes made to the manuscript point-by-point.

**Reviewer 1**

1. I was not aware of the k-mean procedure used by the authors. I find it very interesting. Are you confident that the k-means procedure can be reliable with only 11 subjects to cluster data in a relevant way? If you think so, maybe you could indicate why in the manuscript. Please detail this procedure in the paper: have you pooled all data together or have you repeated the procedure for each different variable? If it was the latter, were the 2 removed subjects clustered together for each variables or only a few? If the latter was the case, this may not be very convincing. To note: the shrinkage property of linear mixed models reduces the influence of outliers, so maybe you don’t really need to remove these subjects (but this is a personal observation).

We decided for k-means clustering, because it is a simple but popular unsupervised machine learning algorithms, making sure data is clustered according to similarity once the number of clusters has been defined. Following the Reviewers suggestion, we provided more information regarding the procedure of cluster analysis. To answer the Reviewer question, data has been pooled together in order to analyses clusters (Line 267 – 274).

2. The statistical models are not sufficiently described by the authors. The authors said they have maximized the models but in the statistic section, it is written that the only random effect was “subject” (while one would expect random slopes and intercepts for each factor and interaction between factors). Similarly, in the result section you display interactions effects (e.g. Light Touch x Test), while these interaction effect are not described in the statistic section. Please indicate clearly the full model equations e.g. Dependent Variable ~ Factor1*Factor2*…+(Factor1*Factor2*…|Subject). Adding random effects for the interactions (as done in the model here) is important, otherwise the type I error rate may go through the roof (see Barr 2013). If you already did all that, please just add all the details in the adequate section.

Full model equations have been added to the statistics section. However, it remains true that only subject is treated as a random factor. All other factors are treated as fixed effects. To our knowledge, this is a valid way of designing and building linear mixed model (Line 279 – 305).

3. The post hoc analyses are not well described and results should be indicated on the corresponding figures to help the reader understand them. Given that you made observations per block, and that your initial models are also dependent of block levels, it seems fair to convey these post hoc analyses also per block level and not just pre vs post as you have done, otherwise there is no interest in using mixed models. But see my last comment before addressing this point (analysis should be done on the EMG normalized to baseline). As a side note, with the type of statistical analysis you have used, you can access model estimates with the summary(model) function. These estimates often make post hoc tests useless since they often answer the required questions, and more importantly for the present case, indicate from where the interaction stems. Displaying these estimates are in general much more informative than the p-values obtained with the function anova (model).

There seems to be a small confusion regarding our models. Our initial model is based on pre vs. post and not block. That is also the reason we decided to add a post hoc test, in order to clarify the effects especially of the stimulation protocol. We tried to describe the post hoc analyses in a more concise and clear matter, in order to make this connection clearer. Only in our second model did we analyze the progression over time (block). However, also for this model did we add a post hoc test, in order to investigate the block differences, especially in the first 4 blocks, as the Reviewer suggested. We did not report model estimates at first, because in our opinion it would flood the manuscript with too many tables. However, due to the Reviewer’s comments we decided to add the model estimates to the appendix, in order to make them available for those interested (Line 300 – 304).

4. I am not sure how you obtained your coefficients in table 4 given the models you have used. You have several fixed effects as factors and therefore you should have a coefficient per factor and per level (plus the interaction coefficients). You can display these coefficients with the function summary(model).

Thanks to the Reviewers comment we realized that this coefficient table was a left over from a previous analysis that we excluded in the final version of manuscript. This table should have been removed as well. We removed the table form the manuscript, since we did not discuss nor reference it anymore and it became obsolete.

5. I think it is a very good idea to normalize EMG values to baseline. Then, you have to redo the tests on the normalized data, which maybe have normal residuals without log transformation. In this case, post hoc analysis must focus on between condition difference at each block level (and please, indicate the results of these post hoc tests on the figures). If significant differences between conditions are observed in blocks performed before the cTBS treatment, you should definitely indicate to the reader in both the abstract and discussion that EMG data variability prevents any clear observation of the effect of cTBS on EMG. If this is the case, this could partly explain why the changes in EMG are not accompanied by behavioral changes during the balance task. If there is no difference in EMG pre cTBS, then you will have a much stronger support for your hypothesis.

We analyzed the data with the normalized EMG values as suggested by the Reviewer. Effects of the stimulation protocol for the Gastrocnemius disappeared but became stronger and clearer for the Tibialis Anterior. We changed the tables and figures accordingly. As the reviewer suggested we also looked at the block differences, specifically in the time before stimulation. We added the post hoc test to the statistics and result section. Even though we find effects of block and touch, no differences in regard to stimulation session are present.

**Reviewer 1**

1. The authors state that the data will be made available. I was not able to access at the time of the revision.

Data will be made available once the Manuscript has been published, as it is stated in the data availability policy.

2. Line 49: “Furthermore, we saw gradual decrease of muscle activity,” upon what?

Sentence has been improved with additional information (Line 50).

3. Line 269: some spaces are missing.

Spaces have been added

4. Line 562: reference style: [Park et al., 2004[40]]

Reference style has been corrected.
